# 918. 2019 Implementation of Universal Hepatitis C Screening at an Urban Federally Qualified Health Center: A Descriptive Analysis and Lessons Learned

**DOI:** 10.1093/ofid/ofab466.1113

**Published:** 2021-12-04

**Authors:** Deborah A Kahal, Karla A Testa, Neal Goldstein

**Affiliations:** 1 Christiana Care Health System, Media, Pennsylvania; 2 Westside Family Healthcare, Wilmington, Delaware; 3 Drexel University Dornsife School of Public Health, Philadelphia, PA

## Abstract

**Background:**

Hepatitis C infection (HCV) is a curable disease that can be effectively managed by non-specialists. Delaware has high HCV rates but limited resources to care for individuals with HCV. Successful HCV micro-elimination starts with universal HCV screening and case identification.

**Methods:**

ChristianaCare (CC) and Westside Family Healthcare (WFH), Delaware’s largest federally qualified health center (FQHC), created a multidisciplinary initiative to support comprehensive HCV care from July 2018-2020 (Figure 1). As part of this partnership, universal opt-out HCV screening in eligible (no prior HCV RNA result) adults ≥ 18 years was implemented at a pilot site in Wilmington in 2019. To characterize screening practices, pre- (risk-based screening) and post-intervention (universal screening) electronic health record data was collected following the first 6 months of the intervention (Jan-June 2019). An HCV dashboard was created and updated monthly to evaluate trends in 2019 screening rates. Collaboration was supported through a 2-year CC Harrington grant.

Figure 1. Components of Federally Qualified Health Center HCV Medical Care Model

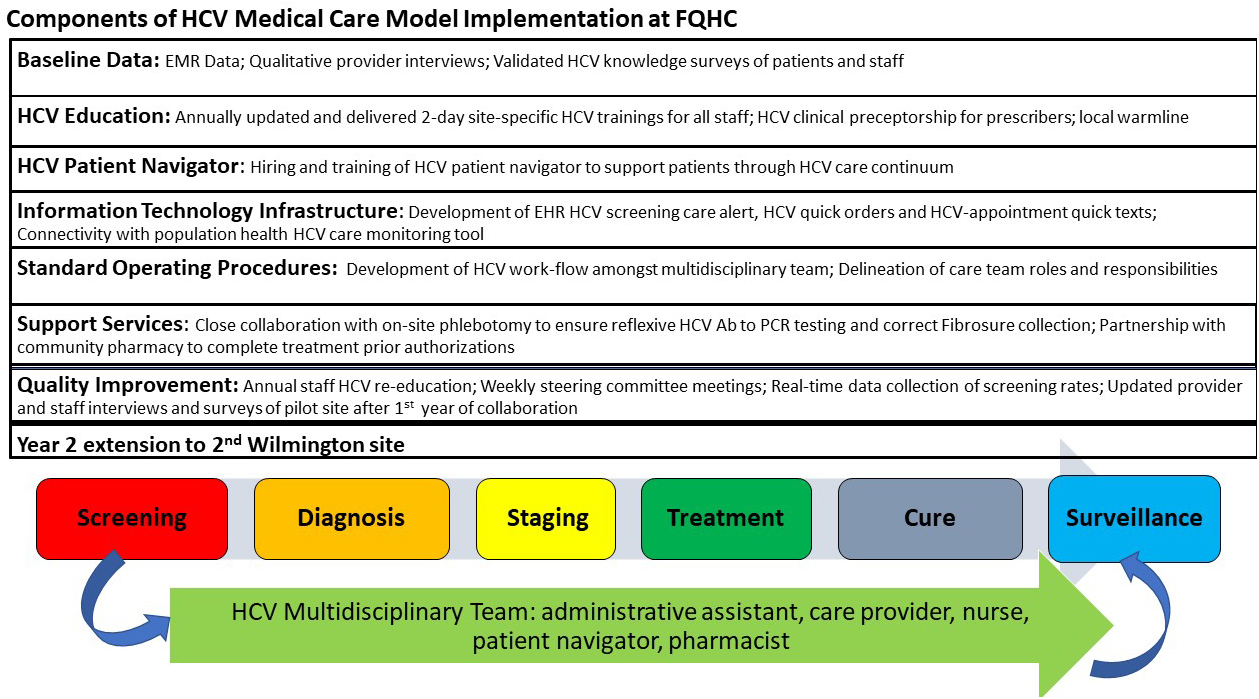

Table 1. Pilot Site Patient Characteristics

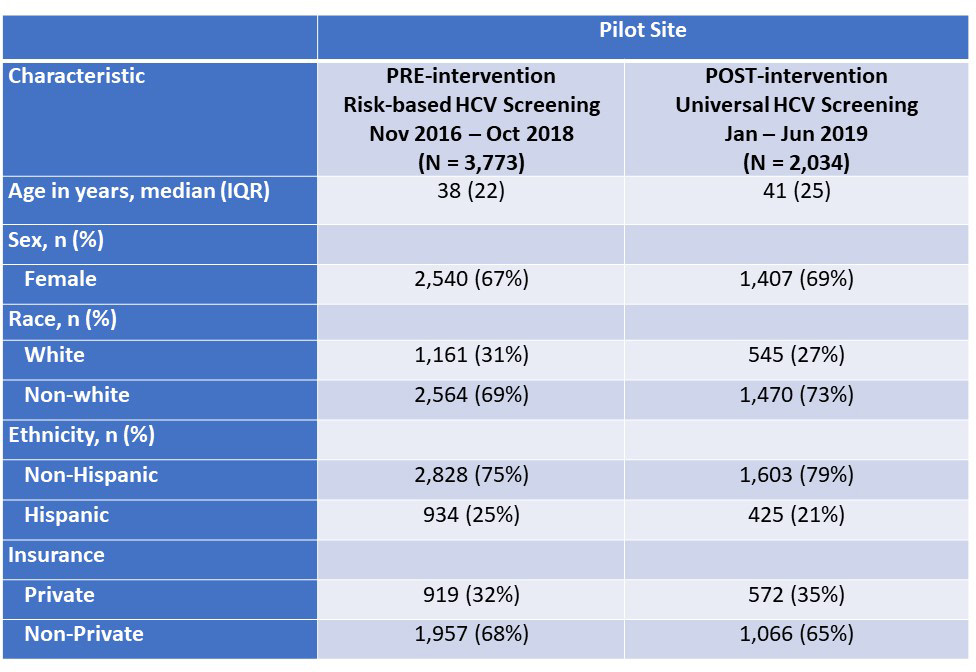

**Results:**

Pre- and post-intervention patient characteristics and screening data are presented in Table 1 and Figure 2 respectively. 39% of patients had screening ordered during the first 6 months of universal screening, a 4% increase from baseline. HCV seroprevalence [amongst resulted tests] remained unchanged from baseline at 5%. During the universal screening period, 2.5% (12/482) of individuals with resulted tests had HCV compared to 4.0% (29/795) tested during risk-based screening. HCV dashboard data demonstrated a trend of increased ordering and fulfillment of screening tests (Figure 3).

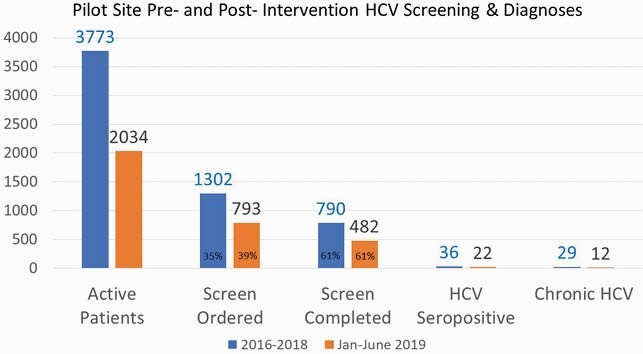

Figure 3. 2019 HCV Dashboard

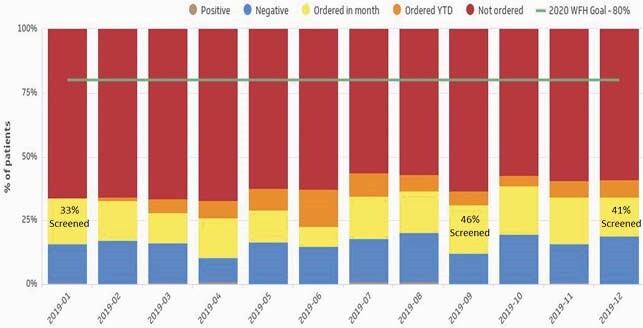

**Conclusion:**

The early adoption of universal HCV screening in adults (prior to 2020 USPSTF update) at an urban FQHC, together with an initiative to provide multidisciplinary HCV care at this FQHC (Figure 1), led to increasing rates of ordered screening. The presented 6-month data does not fully account for lag times between test ordering and fulfillment, resulting in under-reporting of universal HCV screening rates. Multidisciplinary care models to address HCV in patients’ medical homes are vital to HCV eradication with the robust implementation of universal HCV screening a vital first step in this continuum.

**Disclosures:**

**Deborah A. Kahal, MD,MPH, FACP**, **Gilead** (Speaker’s Bureau)**Viiv** (Speaker’s Bureau)

